# Love, Compassion, and Personality as Predictors of Burnout in Nurses: A Path Analysis Study

**DOI:** 10.3390/healthcare14030404

**Published:** 2026-02-05

**Authors:** Agapi L. Batiridou, Elena Dragioti, Zoe Konstanti, Stefanos Mantzoukas, Mary Gouva

**Affiliations:** 1 Scientific Laboratory of Psychology and Person-Centered Care, Department of Nursing, School of Health Sciences, University of Ioannina, 45500 Ioannina, Greece; batiridou@uoi.gr (A.L.B.); dragioti@uoi.gr (E.D.); zkonstanti@uoi.gr (Z.K.); 2 Qualitative Research & Reflective Based Nursing Praxis, Department of Nursing, School of Health Sciences, University of Ioannina, 45500 Ioannina, Greece; smantzoukas@uoi.gr

**Keywords:** burnout, compassion, depersonalization, emotional exhaustion, extraversion, love, neuroticism, nurses, path analysis, personality traits

## Abstract

**Highlights:**

**What are the main findings?**

- Compassion is negatively associated with burnout, while love-related emotions show additional but more limited associations with burnout dimensions.
- Personality traits are strongly associated with burnout, with higher neuroticism linked to greater burnout levels and positive personality traits associated with lower burnout.

**What are the implications of the main findings?**

- Interventions aimed at enhancing compassion may be relevant for addressing depersonalization and professional accomplishment, thereby potentially supporting nurses’ well-being.
- Organizational strategies, such as improved shift scheduling and attention to individual vulnerability profiles (e.g., higher neuroticism), may be important considerations in efforts to address burnout-related risk factors.

**Abstract:**

**Background/Objectives**: This study examined how personality traits, compassion, and love are associated with the three dimensions of burnout among nurses, while accounting for demographic factors such as gender, age, and work shift. **Methods**: A total of 403 nurses participated in this cross-sectional study and completed validated self-report measures of personality, compassionate love, and burnout, as well as an in-house, exploratory Love Instrument. Path analysis was used to examine patterns of direct and indirect associations among the study variables while controlling demographic covariates. **Results**: Men reported higher psychoticism and depersonalization, whereas women scored higher in compassion. Neuroticism was associated with greater emotional exhaustion and depersonalization and with lower personal accomplishment. Compassion showed indirect association patterns linking extraversion and the Lie scale with personal accomplishment and linking psychoticism with depersonalization. Extraversion was positively associated with accomplishment both directly and indirectly, while psychoticism was associated with higher depersonalization. Love-related variables showed mixed findings. Specifically, love experience was not associated with burnout, whereas love intensity was positively associated with both depersonalization and accomplishment. Older nurses reported more exhaustion but also greater accomplishment; male gender and rotating shifts were associated with higher depersonalization and exhaustion. **Conclusions**: The findings support neuroticism as a key dispositional vulnerability correlated with burnout and suggest that compassion and extraversion are linked to more favorable burnout-related profiles, particularly higher accomplishment and lower depersonalization. Love-related emotion intensity showed small, mixed associations and should be interpreted cautiously given the exploratory measurement approach. These results highlight the emotional complexity of nursing and may inform future research and workplace initiatives aimed at supporting occupational well-being.

## 1. Introduction

Burnout among nurses remains a major occupational health challenge worldwide. It is commonly characterized by emotional exhaustion, depersonalization, and reduced personal accomplishment, and is associated with adverse outcomes for both healthcare professionals and patient care [1,2]. Recent systematic reviews and meta-analyses indicate that moderate to high levels of burnout are prevalent in nursing, particularly in emotionally demanding settings such as intensive care, oncology, psychiatric services, and long-term or palliative care [3,4,5,6,7]. Despite increased organizational attention, burnout remains a persistent and multifactorial problem.

In seeking to explain this persistence, contemporary frameworks conceptualize burnout as a response to chronic occupational stressors, including high workload, time pressure, low job control, inadequate staffing, and limited organizational support [8,9,10,11]. However, similar working conditions do not affect all nurses in the same way, highlighting the role of individual differences in stress appraisal and emotional responding [2,12]. In parallel, recent European studies underscore the importance of emotional resources and emotional functioning for burnout-related outcomes in nursing populations, including the role of student nurses as the future workforce [13,14].

These observations have directed attention toward more stable individual characteristics that may shape how nurses engage with their work emotionally. Personality traits represent relatively stable dispositional factors that influence emotional reactivity and coping tendencies. Across broad trait frameworks, higher neuroticism is consistently associated with higher burnout, whereas extraversion and other adaptive traits are generally linked to lower burnout and greater work engagement [15,16,17,18,19]. Importantly, personality is best understood as a distal predisposition rather than a direct cause of burnout, with its associations likely operating through emotional and psychosocial mechanisms [16,19,20,21].

Beyond dispositional factors, the emotional nature of nursing work itself plays a central role in shaping well-being. Emotional engagement is fundamental to nursing practice. Compassion, defined as sensitivity to suffering coupled with a motivation to alleviate it, has been described as a regulated and ethically grounded professional emotion [22,23,24]. When supported by effective emotion regulation strategies and adequate organizational resources, compassion may buffer the impact of chronic stress [23,25]. In contrast, when such support is insufficient, sustained exposure to suffering may contribute to compassion fatigue and emotional exhaustion [26,27,28]. Qualitative accounts also describe the emotional cost of sustained caregiving under chronic fatigue, high workload, and limited resources [29].

Although compassion has received substantial empirical attention, nurses’ emotional experiences are not confined to compassion alone. In everyday practice, nurses may also experience broader love-related emotions, such as emotional closeness, attachment, and deep relational involvement with patients, families, or colleagues [30,31,32]. These experiences have received comparatively less attention in nursing research, even though qualitative and conceptual studies suggest that nurses often describe caring in terms that go beyond regulated compassion, using expressions such as “loving like family,” emotional closeness, and personal involvement with patients and relatives [33,34].

In the present study, love-related emotions are introduced as an exploratory construct intended to capture the depth and intensity of relational bonding, rather than as a validated caregiving mechanism. This approach is consistent with emotional labor research, which has long acknowledged the central role of emotional involvement in nursing and therapeutic relationships, while also noting that such experiences remain only partially theorized and empirically measured [32,35].

Distinguishing between different forms of emotional engagement is therefore important in this context. Compassion is typically conceptualized as an ethically regulated, role-consistent response aimed at alleviating suffering. Love-related emotions, by contrast, may be more diffuse and encompass attachment, emotional closeness, and identification with patients or colleagues [30,32]. Existing evidence suggests that this type of intense emotional involvement can be ambivalent in its effects. When supported by emotional competence and appropriate professional boundaries, it may enhance meaning, satisfaction, and perceived quality of care. At the same time, in the absence of sufficient organizational and regulatory support, it may increase emotional labor, vulnerability to burnout, and difficulties disengaging from work [31,35,36].

**Figure 1:**
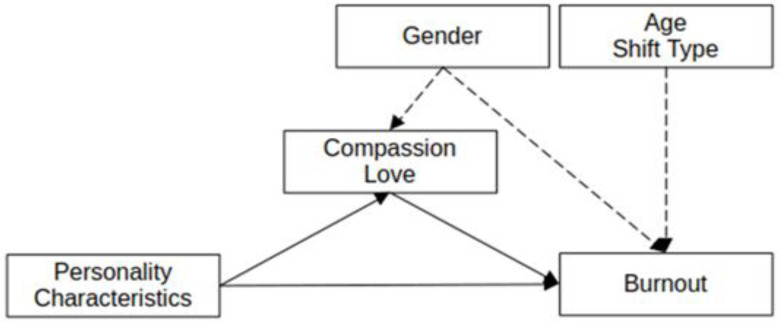
Structural Path Model. Compassion and love-related emotions were modeled as parallel mediators. Age, gender, and shift type were included as covariates (controls). The dashed lines indicate covariates and the solid lines indicate parallel mediators.

Taken together, this work points to a complex interplay between dispositional characteristics and forms of emotional engagement in nursing. Personality traits may shape nurses’ emotional tendencies, while compassion and love-related emotions represent distinct modes of engagement with potentially different implications for burnout. In light of this framework, the present study conceptualizes compassion and love-related emotions as parallel emotional pathways, rather than sequential stages, through which dispositional characteristics may be statistically associated with burnout outcomes. Using an exploratory path-analytic approach, the study aims to examine patterns of association among personality traits, compassion, love-related emotional experiences, and burnout (Figure 1). Given the cross-sectional design and the exploratory status of the love-related construct, the goal is to describe relationships and generate hypotheses rather than to draw causal conclusions. Accordingly, the study addresses three central research questions:

- Are compassion and love-related emotions associated with the relationships between personality traits and burnout outcomes (i.e., do they function as potential mediating pathways in the model)?
- How do these indirect association patterns differ across the three burnout dimensions?
- Are demographic and work-related factors (age, gender, and shift type) associated with differences in these pathways among nurses?

## 2. Materials and Methods

### 2.1. Participants and Procedure

Data collection for this cross-sectional study was conducted during the spring and summer of 2024. A total of 418 nurses (342 women and 76 men) participated. A comprehensive questionnaire was administered by the first author of the present manuscript. Following prior telephone communication with unit supervisors/administrators to facilitate distribution, the questionnaire was distributed both electronically, via a link created using Google Forms, and in paper format. The online form did not collect identifying information (e.g., names, emails, or IP addresses), and responses were recorded anonymously. The paper version was hand-delivered to nurses during their work shifts. The completion of the questionnaire required approximately 35 min. Participants completed the questionnaire either during breaks within their working hours or outside their working schedule. Completed paper questionnaires were returned in sealed envelopes to ensure confidentiality.

Eligibility criteria required at least one year of professional nursing experience and current employment in General or University hospitals, Primary Health Care Centres, or other healthcare facilities providing clinical care in Greece. Participation was strictly voluntary and anonymous, and no financial or material incentives were provided. No performance-related conditions, managerial pressure, or employment-related consequences were linked to participation. Participants were informed that participation or non-participation would not affect their work evaluation or duties. Exclusion criteria included nurses who were not actively employed during the study period or who did not wish to participate voluntarily.

Of the initial sample, eight respondents did not report their age and seven did not provide marital status information; these fifteen cases were excluded from subsequent analyses. Thus, the final sample consisted of 403 nurses (331 women and 72 men), with a mean age of 43.2 years (SD = 10.4). The majority were married (61.8%, n = 249), had more than five years of professional experience (76.2%, n = 307), and worked rotating shifts (53.3%, n = 215).

### 2.2. Measures

Participants completed a comprehensive questionnaire comprising validated psychometric instruments. These included the Santa Clara Brief Compassion Scale (SCBCS; Santa Clara University, Santa Clara, CA, USA) [37], the Maslach Burnout Inventory (MBI; Mind Garden, Inc., Menlo Park, CA, USA) [38], and the Eysenck Personality Questionnaire (EPQ; Hodder & Stoughton, London, UK) [39,40,41].

The SCBCS is a five-item self-report measure of compassion derived from the Compassionate Love Scale [37,42]. It assesses core components of compassion, including empathic concern, tender feelings toward others, and compassionate love conceptualized as altruistic concern for all human beings. Items are rated on a 7-point Likert scale (1–7), yielding total scores ranging from 5 to 35, with higher scores indicating greater compassion. The scale demonstrates excellent internal consistency (Cronbach’s α = 0.89) [37,42].

MBI is a widely used self-report instrument assessing burnout across three dimensions: emotional exhaustion, depersonalization, and reduced personal accomplishment [38]. The scale has demonstrated satisfactory to excellent internal consistency across its subscales (Cronbach’s α = 0.71–0.88), with higher scores indicating greater levels of burnout [43].

The EPQ is a self-report personality questionnaire developed by Eysenck and Eysenck [40] to assess major dimensions of personality, including psychoticism, extraversion, neuroticism, and a Lie (social desirability) scale. The instrument has demonstrated acceptable to good internal consistency across its subscales, with higher scores reflecting greater expression of the corresponding personality traits [40,41].

An in-house Love Instrument (University of Ioannina, Ioannina, Greece) was developed specifically for this study to capture two theoretically related facets of love-related experience in caregiving contexts. Item generation was informed by (a) conceptual definitions of love-related affect and relational involvement in caregiving relationships, and (b) the structure and response format of the SCBCS as a pragmatic template for concise self-report assessment. An initial pool of items was designed to represent two domains: love experience (LES; cognitive–affective appraisal of love as a value and lived experience) and love-related emotion intensity (LREIS; intensity of love-related and relational emotions toward others). Items were reviewed by the research team for clarity, redundancy, and face validity, and were refined through iterative revision. In the present sample, both subscales demonstrated satisfactory internal consistency (Cronbach’s α = 0.92 and 0.87, respectively). As no independent pilot sample was available prior to the main data collection, the Love Instrument is considered exploratory and hypothesis-generating.

The LES (14 items) captures the cognitive–affective aspects of love (e.g., “Love is important for my well-being”, “I experience vitality through loving relationships”), rated on a 7-point scale (1 = strongly disagree to 7 = strongly agree). The LREIS (14 items) evaluates the intensity of emotional responses toward others (e.g., love, passion, trust, jealousy) on a 7-point scale (0–6). Higher scores in both dimensions indicate greater emotional engagement. Although not formally standardized, the instrument demonstrated conceptual coherence and satisfactory internal reliability indices, serving as an exploratory framework for understanding love-related processes in the nursing context.

In addition, the questionnaire collected sociodemographic data such as age, gender, marital status, years of professional experience, and work schedule.

### 2.3. Data Analysis

Standard univariate tests (independent-samples *t*-tests and chi-square tests of independence) were first applied to examine potential effects of gender and shift type on personality traits, compassion toward others, and burnout dimensions. Partial correlations were then computed to explore the unique associations between the independent variables (EPQ-personality traits) and the outcome variables (burnout subscales), controlling for the mediators love experience, love-related emotion intensity and compassion. This step served as a preliminary check to confirm that the proposed mediators were sufficiently related to both the independent variables and outcomes to justify testing mediation effects.

To evaluate the contribution of both love-related scores, i.e., love experience and love-related emotion intensity as variables of interest, a series of nested path models were estimated separately for each variable. For each comparison, a restricted model excluding the variable of interest was contrasted with a full model including it. Chi-square difference tests (Δχ^2^) were used to determine whether the inclusion of each variable significantly improved model fit. Based on these comparisons, both love scales were retained for the final model.

Path analysis was selected to examine a theoretically specified network of direct and indirect associations among observed scale scores and to enable the simultaneous estimation of multiple mediation pathways within a single unified model, which cannot be adequately tested using separate regression analyses. The model tested the hypothesis that compassion and love mediate the relationships between personality traits and burnout.

In the model, the four personality traits were specified as having direct associations with compassion and love, while compassion, love, and personality traits were simultaneously specified as having direct associations with the three burnout subscales. Age and shift type were included as covariates of the burnout dimensions, as their effects are well established in the literature. In contrast, age and shift type were excluded from compassion and love regressions because prior research [44] and the present data indicated no meaningful variation in these constructs across demographic groups; this decision also facilitated model identification.

Model parameters were estimated using maximum likelihood estimation. Model fit was evaluated using the chi-square goodness-of-fit test and commonly reported fit indices, including the Root Mean Square Error of Approximation (RMSEA), Standardized Root Mean Square Residual (SRMR), Tucker–Lewis Index (TLI), and Comparative Fit Index (CFI). All analyses were conducted in R (R Foundation for Statistical Computing, Vienna, Austria; version 4.3.3) [45] using the lavaan package (R Team, Vienna, Austria; version 0.6-20) [46].

As described in the participants and procedure section, missing data was handled using listwise deletion. All subsequent analyses were conducted on the resulting complete-case sample (N = 403).

Potential common method variance was examined at the scale level using a single-factor confirmatory factor analysis, as item-level data were not available. All scale scores were specified to load onto a single latent factor. The model demonstrated poor fit to the data (χ^2^(35) = 323.00, CFI = 0.61, TLI = 0.49, RMSEA = 0.14, SRMR = 0.11), suggesting that a single common method factor is unlikely to account for the observed covariance structure. Multicollinearity among variables was assessed using variance inflation factors (VIFs). All VIF values were close to 1 (extraversion = 1.16, neuroticism = 1.21, psychoticism = 1.12, Lie scale = 1.16, compassion = 1.23, LES = 1.26, LREIS = 1.32), indicating no evidence of multicollinearity.

Construct validity at the scale level was further supported by the pattern of intercorrelations among study variables and by theoretically consistent associations observed in the path analysis, providing evidence of both discriminant and nomological validity.

## 3. Results

### 3.1. Descriptive Statistics

Descriptive statistics and Pearson correlation coefficients for all study variables are presented in Table 1. Burnout subscale scores for emotional exhaustion (M = 25.1, SD = 12.0), depersonalization (M = 8.39, SD = 6.27), and personal accomplishment (M = 36.0, SD = 6.61). Participants showed comparable levels of extraversion (M = 13.5, SD = 4.20) and neuroticism (M = 10.9, SD = 4.70), and lower psychoticism scores (M = 4.84, SD = 2.05). No significant differences were observed across gender, marital status, or shift type for most study variables, and gender and shift type were independent, χ^2^(1) = 0.012, *p* = 0.914.

No significant gender differences were observed for love experience, love-related emotion intensity, extraversion, neuroticism, the Eysenck Lie subscale, emotional exhaustion, or personal accomplishment. Similarly, love experience and love-related emotion intensity did not differ significantly between nurses working rotating versus fixed shifts. Significant gender differences were observed for psychoticism, depersonalization, and compassion, with men scoring higher on psychoticism and depersonalization, and women reporting higher compassion.

Bivariate correlations indicated that compassion was associated with lower depersonalization and higher personal accomplishment, whereas love-related variables showed generally small associations with burnout dimensions (Table 1). Preliminary partial correlations were examined to justify inclusion of compassion and love-related variables as mediators and are reported for completeness in the Supplementary Materials (Figure S1).

Nested model comparisons were conducted to evaluate the contribution of love experience and love-related emotion intensity to model fit (Table S1). The full model, including compassion, love experience, and love-related emotion intensity as parallel mediators, demonstrated superior fit and was therefore retained for subsequent analyses.

### 3.2. Path Analysis Results

Based on these preliminary analyses, subsequent results focus on the final path model examining compassion and love-related emotion intensity as parallel emotional pathways linking personality traits to burnout. The final model demonstrated excellent fit to the data, χ^2^(6) = 5.84, *p* = 0.441, with fit indices indicating very good model adequacy (CFI = 1.00, TLI = 1.00, RMSEA = 0.00, SRMR = 0.010).

The model explained 5.8% of the variance in love-related emotion intensity, 6.3% in love experience, 11.8% in compassion, 31.6% in emotional exhaustion, 24.8% in depersonalization, and 24.3% in personal accomplishment. Significant direct effects are summarized in Table 2, and significant indirect and total effects are presented in Table 3 (full results are available in Tables S2 and S3). Standardized coefficients indicated that neuroticism showed the strongest association with emotional exhaustion (Std. All = 0.50), whereas love-related emotion intensity exhibited small but significant associations with depersonalization (Std. All = 0.10) and personal accomplishment (Std. All = 0.16).

### 3.3. Direct Effects

Love-related emotion intensity was positively associated with depersonalization (b = 0.10, *p* = 0.043) and personal accomplishment (b = 0.17, *p* = 0.001) and was not associated with emotional exhaustion (*p* = 0.443). Love experience showed no significant direct associations with any burnout dimension.

Emotional exhaustion was strongly associated with neuroticism (b = 0.50, *p* < 0.001). No significant associations were observed for love experience (*p* = 0.360), love-related emotion intensity (*p* = 0.443), compassion (*p* = 0.736), extraversion (*p* = 0.539), psychoticism (*p* = 0.103), or the Eysenck Lie subscale (*p* = 0.599). Age (b = 0.13, *p* = 0.004) and rotating shift work (b = 0.14, *p* = 0.001) were positively associated with emotional exhaustion.

Depersonalization was negatively associated with compassion (b = −0.13, *p* = 0.007) and positively associated with neuroticism (b = 0.27, *p* < 0.001), psychoticism (b = 0.24, *p* < 0.001), and rotating shift work (b = 0.13, *p* = 0.003). Gender (*p* = 0.054) and age (*p* = 0.969) were not significantly associated with depersonalization; however, the total effect of gender was significant, reflecting an indirect association via compassion (Table 3).

**Figure 2:**
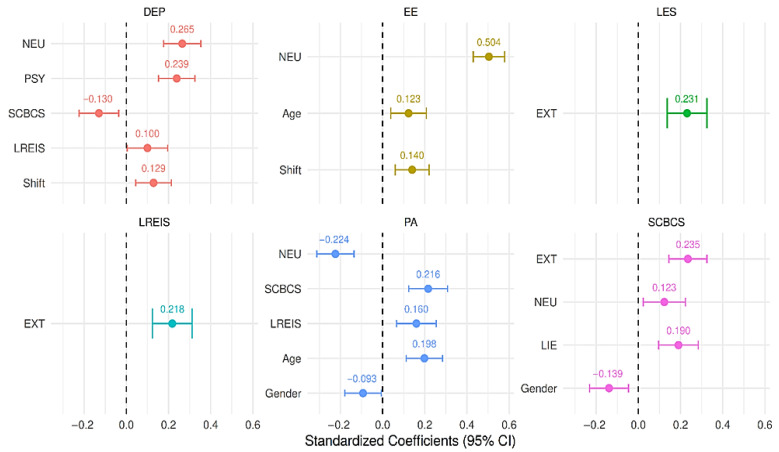
Significant direct pathways from the path analysis model.

Personal accomplishment was positively associated with love-related emotion intensity (b = 0.11, *p* = 0.001), compassion (b = 0.22, *p* < 0.001), and age (b = 0.20, *p* < 0.001), and was negatively associated with neuroticism (b = −0.22, *p* < 0.001). Shift type was not significantly associated with personal accomplishment (*p* = 0.899). A significant gender difference was observed, with women reporting higher personal accomplishment (b = −0.09, *p* = 0.034). Significant associations are illustrated in Figure 2. Overall, effects involving love-related emotion intensity were statistically significant but small in magnitude.

### 3.4. Assessment of the Mediating Role of Love and Compassion Variables

Examination of indirect and total effects indicated distinct roles for compassion, love experience, and love-related emotion intensity across the burnout dimensions. The LES showed no significant indirect or total effects on emotional exhaustion, depersonalization, or personal accomplishment, indicating an absence of both mediation and direct associations across all burnout dimensions.

Compassion demonstrated significant indirect associations between personality traits and burnout outcomes. Higher extraversion and lower neuroticism were associated with greater personal accomplishment through compassion (extraversion: indirect b = 0.08, *p* = 0.001; neuroticism: indirect b = 0.04, *p* = 0.034). In addition, extraversion, the Eysenck Lie subscale, and gender were indirectly associated with depersonalization via compassion.

To aid interpretation, standardized coefficients were evaluated by relative magnitude rather than strict practical thresholds. Coefficients of |β| ≈ 0.10 were considered small, |β| ≈ 0.30 moderate, and |β| ≥ 0.50 relatively strong. Analysis of standardized total effects showed that the association between neuroticism and emotional exhaustion was the largest effect in the model (Std. All ≈ 0.50), while indirect effects via compassion were smaller but statistically significant for depersonalization and personal accomplishment.

The LREIS did not show indirect associations with emotional exhaustion or personal accomplishment but demonstrated several significant total associations. Extraversion was associated with personal accomplishment through love-related emotion intensity (total b = 0.162, *p* = 0.002), with similar total associations observed for neuroticism, psychoticism, and the Eysenck Lie subscale. Depersonalization showed a modest total association with extraversion via love-related emotion intensity (total b = 0.096, *p* = 0.046). Overall, love-related emotion intensity was associated with burnout primarily through total rather than indirect effects.

## 4. Discussion

This study used an exploratory path-analytic framework to examine how broad personality traits, compassion, and love-related emotions are associated with the three burnout dimensions among Greek nurses. While our previous studies examined burnout-related outcomes in Intensive Care Unit healthcare professionals using different analytic approaches, the present study focuses specifically on nurses across healthcare settings and tests an expanded, exploratory model that differentiates compassion from love-related emotion intensity as parallel pathways. Importantly, the present analyses are based on a new and independent dataset and address distinct research questions not examined in our prior publications [47,48]. Neuroticism showed the strongest and most consistent associations with burnout, while rotating shift work and age were also linked to less favorable burnout profiles. Compassion was associated with lower depersonalization and higher personal accomplishment, whereas love-related variables showed mixed and generally small associations, with love experience largely non-significant.

From a theoretical standpoint, the findings are consistent with an interplay between dispositional tendencies, emotional engagement, and work conditions in relation to burnout. From occupational stress and emotion-regulation perspectives, burnout may be understood as reflecting sustained demands that exceed coping and recovery resources. In this context, traits linked to negative affectivity and stress sensitivity typically show stronger associations with exhaustion and depersonalization [49], whereas regulated prosocial emotions such as compassion may relate more closely to interpersonal functioning and professional meaning [25,50]. Love-related emotion intensity—examined here with an exploratory measure—may reflect broader relational involvement that can be experienced ambivalently, potentially supporting meaning while also being linked to greater emotional labor and difficulty detaching from work [51,52,53]. Against this conceptual background, the present model helps situate these pathways within a single integrative framework.

These findings add nuance to review-level evidence by illustrating how dispositional characteristics and differentiated forms of emotional engagement can co-occur within a single explanatory framework, rather than appearing as isolated correlates of burnout [7,8,10]. Consistent with qualitative accounts, the results also underscore the centrality of emotional and relational demands in nurses’ experience of burnout; however, the present model suggests that emotional involvement is not uniformly associated with unfavorable outcomes, highlighting the importance of distinguishing regulated compassion from more intense relational emotionality [29,54,55].

Neuroticism was associated with less favorable profiles across the burnout dimensions, consistent with evidence linking heightened emotional reactivity and stress sensitivity to burnout-related outcomes in nursing populations [18,49,56,57]. Psychoticism was also associated with higher depersonalization, suggesting that emotionally distancing interpersonal styles may co-occur with disengagement under occupational strain [47]. In contrast, extraversion was associated with higher personal accomplishment, both directly and indirectly through compassion, aligning with prior work indicating that socially adaptive and approach-oriented traits are related to engagement and perceived effectiveness at work [58,59,60]. Taken together, these findings suggest that personality-related associations remain evident even when emotional variables are considered concurrently, supporting the view that dispositional tendencies are embedded within broader emotional processes [16,17,61]. Consistent with mediation-oriented perspectives, compassion and related emotional resources may function as relational pathways linking personality to burnout-relevant outcomes [62,63]. This pattern is broadly consistent with occupational stress and emotion-regulation accounts, which conceptualize burnout as emerging when sustained demands exceed available coping and recovery resources and where individual differences in emotional reactivity shape stress experiences at work [2,8,12]. Within this framework, compassion aligns with lower depersonalization and higher personal accomplishment, whereas love-related emotion intensity—treated here as exploratory—may capture ambivalent emotional involvement linked to both perceived meaning and increased emotional labor and detachment difficulties [26,27,32,34,35,59], which may help explain its mixed associations across burnout dimensions.

Work and demographic factors were also meaningfully associated with burnout. Older nurses reported higher emotional exhaustion alongside higher personal accomplishment, suggesting that cumulative occupational strain may coexist with increased mastery and professional meaning over time [64]. Rotating shift work was linked to higher emotional exhaustion and depersonalization, consistent with evidence that irregular schedules disrupt sleep, recovery, and circadian regulation and are associated with greater burnout vulnerability in healthcare workers [65,66]. Taken together, these findings suggest that individual emotional resources may not fully counterbalance sustained system-level strain, aligning with broader evidence that burnout in healthcare professionals reflects multiple contextual and organizational determinants [7,67,68]. Recent umbrella reviews and longitudinal syntheses similarly highlight shift work and workload-related fatigue as among the most robust occupational correlates of emotional exhaustion, often showing stronger associations than individual psychological resources [7,9,10].

Gender-related findings should be interpreted cautiously. Men reported higher psychoticism and depersonalization, whereas women reported higher compassion and personal accomplishment. Within nursing contexts, such differences may reflect gender-role expectations and emotion-management norms, including distinct pressures on male nurses regarding emotional expression and interpersonal engagement [69]. More broadly, gender role expectations can shape empathy-related responses [70], and meta-analytic evidence suggests that gender differences in burnout are context-dependent rather than uniform across settings [71]. However, the relatively small male subsample limits the precision of these estimates; therefore, gender-related patterns should be considered exploratory.

Compassion showed a differentiated pattern across burnout dimensions. It was unrelated to emotional exhaustion but was linked to lower depersonalization and higher personal accomplishment, suggesting closer relevance to interpersonal connection and professional meaning than to fatigue-driven depletion [25,50]. This pattern aligns with cross-sectional evidence in nursing populations indicating that compassion-related resources are more strongly associated with relational functioning and perceived professional efficacy than with exhaustion per se [72,73,74]. Indirect association patterns further suggested that personality-linked resources (e.g., extraversion and social desirability) were associated with depersonalization and personal accomplishment through compassion, supporting a resource-oriented pathway described in healthcare contexts [75]. Placed alongside review-level evidence, these findings suggest that compassion may be more relevant to relational and self-evaluative aspects of burnout, whereas emotional exhaustion appears more closely tied to workload, sleep disruption, and recovery deficits [3,76]. Consistent with this interpretation, intervention-focused reviews indicate that compassion-related skills may be insufficient as standalone approaches and may be most effective when combined with emotion-regulation support and organizational-level actions [77,78]. In line with evidence, the present findings further suggest that emotion-related processes are relevant to burnout-related well-being, extending prior work in nursing students to practicing nurses and highlighting the potential continuity of emotional pathways across career stages [13,14].

Findings related to love-related emotions were mixed and warrant cautious interpretation. Love experience showed no meaningful direct or indirect associations with burnout outcomes in the final model, whereas love-related emotion intensity displayed small positive associations with both depersonalization and personal accomplishment. This pattern suggests that intense relational involvement may co-occur with higher professional meaning and satisfaction while also being linked to emotional distancing in certain contexts—potentially as a coping response when demands are high or professional boundaries are difficult to maintain. Such ambivalence is consistent with conceptual accounts of love and emotional closeness in nursing and therapeutic relationships [51,52,53], as well as with broader evidence that relational strain and relational support can exert opposing influences on well-being [79].

Several alternative explanations should be considered. First, weak and mixed associations may reflect measurement limitations given the exploratory status of the Love Instrument. Second, conceptual overlap with compassion and related emotional constructs may have reduced unique associations [25,50]. Third, love-related processes may operate through pathways not captured in the present analyses, such as boundary management, emotion regulation strategies, compassion fatigue, or secondary stress processes [76,80,81]. Consistent with this view, reviews of emotional labor in nursing suggest that heightened affective intensity can enhance perceived care quality while simultaneously increasing vulnerability to emotional strain and detachment when regulatory resources are limited [30,32,33,35,36]. Taken together, these findings suggest that love-related emotion intensity occupies a more ambiguous position relative to burnout than compassion and should be regarded as hypothesis-generating. Replication with validated measures and longitudinal designs is needed to clarify whether love-related emotion intensity represents a distinct construct or reflects overlapping processes within emotional labor and relational engagement.

### 4.1. Clinical and Practical Implications

A practical contribution of the present model lies in its differentiation between (i) compassion, which is associated with lower depersonalization and higher personal accomplishment, and (ii) love-related emotion intensity, which shows mixed associations and may reflect strain related to emotional boundaries. From a nursing management perspective, these findings highlight the value of a dual approach: strengthening regulated compassion to support interpersonal connection and professional fulfillment, while simultaneously promoting boundaries and recovery processes to reduce emotional overextension.

The magnitude of the observed effects further clarifies the applied significance of the model. The strongest associations were observed for neuroticism and compassion, suggesting that even relatively modest differences in emotional stability and compassion-related resources may translate into meaningful differences in daily work strain, emotional distancing, and professional fulfillment. In high-demand clinical environments, such effects are likely to accumulate over time, thereby amplifying their relevance for nurses’ well-being.

By contrast, the effect sizes associated with love-related constructs were generally small. Love experience showed consistently negligible associations across burnout dimensions, whereas love-related emotion intensity demonstrated modest and mixed associations, potentially enhancing professional accomplishment while also increasing vulnerability to depersonalization. Accordingly, although statistically detectable, these effects appear to play a secondary role relative to personality traits and compassion when considering burnout prevention.

In practical terms, these findings suggest that interventions may benefit from combining compassion-focused skill development with boundary-setting and emotion-regulation strategies. Reflective supervision and structured peer debriefing may further support recovery, particularly for staff with higher stress sensitivity (e.g., elevated neuroticism). In parallel, organizational measures that protect recovery time—especially for nurses working rotating shifts—remain essential, given the established links between shift-related fatigue and burnout risk [66]. Interventions targeting compassion and professional quality of life may therefore be most effective when implemented alongside structural improvements in staffing and scheduling [7,66].

### 4.2. Limitations and Future Directions

Several limitations should be acknowledged. First, the cross-sectional design precludes causal inference; the reported paths reflect associations, and indirect effects should be interpreted as statistical patterns consistent with mediation rather than temporal mechanisms. Second, self-report measures may introduce common-method variance and social desirability influences.

Third, the use of convenience sampling entails inherent limitations for external validity, as participants were recruited through non-random procedures and may not be representative of the broader nursing population. Selection bias cannot be ruled out, and the strength or direction of observed associations may differ in samples drawn from other healthcare settings, specialties, or organizational contexts. In addition, the underrepresentation of male nurses further limits generalizability, particularly for gender-related effects, as emotional engagement and burnout processes may vary across roles, units, and institutional cultures [82]. Accordingly, findings should be interpreted as context-specific rather than population-level estimates.

A key limitation concerns the in-house Love Instrument. Although internal consistency was satisfactory in the present sample, the instrument was not evaluated in an independent pilot sample prior to the main survey and has not undergone full psychometric validation. Future studies should test its factor structure, examine convergent and discriminant validity against established compassion and emotional functioning measures, and assess test–retest reliability in independent samples [44,58]. Longitudinal, multi-site, and cross-cultural designs are also needed to examine temporal ordering and clarify whether love-related emotion intensity represents a distinct construct or overlaps with compassion and emotional labor processes [25,50,51]. Accordingly, findings involving love-related emotions should be considered hypothesis-generating and interpreted cautiously.

Finally, using the EPQ rather than a Five-Factor instrument may limit direct comparability with some studies; however, core domains such as neuroticism and extraversion show conceptual continuity across personality frameworks, supporting interpretability within the broader trait literature [83,84,85].

## 5. Conclusions

In this cross-sectional sample of nurses, neuroticism showed the most consistent links with burnout, supporting its role as a dispositional vulnerability marker. Compassion was associated with lower depersonalization and higher personal accomplishment and showed indirect association patterns linking personality to these outcomes, suggesting that regulated prosocial engagement may be particularly relevant to relational and meaning-related aspects of burnout. Love experience showed no clear association with burnout, whereas love-related emotion intensity exhibited small and mixed associations with depersonalization and personal accomplishment, underscoring the need for cautious interpretation and further validation before applied conclusions are drawn. Overall, the findings suggest that nurse well-being reflects an interplay of dispositional tendencies, emotional engagement, and organizational conditions, and that workplace initiatives may benefit from combining compassion-related support with structural strategies that protect recovery, particularly for nurses working rotating shifts.

## Figures and Tables

**Table 1:** Descriptive Statistics and Pearson Correlation Matrix for Study Variables.

	M (SD)	Age	SCBCS	LES	LREIS	MBI			EPQ		
						EE	DEP	PA	EXT	NEU	PSY
SCBCS	27.8 (4.95)	0.098 *									
LES	72.1 (14.0)	0.034	0.246 ***								
LREIS	53.0 (9.88)	0.030	0.324 ***	0.420 ***							
MBI											
EE	24.9 (11.9)	0.034	−0.044	−0.103 *	−0.098						
DEP	8.38 (6.29)	−0.095	−0.176 ***	−0.123 *	−0.013	0.593 ***					
PA	36.0 (6.61)	0.253 ***	0.339 ***	0.223 ***	0.294 ***	−0.201 ***	−0.255 ***				
EPQ											
EXT	13.6 (4.17)	0.106 *	0.223 ***	0.231 ***	0.224 ***	−0.147 **	−0.139 **	0.230 ***			
NEU	10.9 (4.64)	−0.101 *	0.008	−0.061	−0.084	0.526 ***	0.346 ***	−0.283 ***	−0.219 ***		
PSY	4.83 (2.06)	−0.110 *	−0.101 *	−0.060	−0.046	0.211 ***	0.354 ***	−0.159 **	−0.031	0.258 ***	
LIE	14.9 (3.51)	0.232 ***	0.192 ***	0.012	0.046	−0.154 **	−0.216 ***	0.200 ***	0.070	−0.277 ***	−0.237 ***

Note: *: *p* < 0.05, **: *p* < 0.01, ***: *p* < 0.001 SCBCS = Santa Clara Brief Compassion Scale; LES = Love Experience Scale; LREIS = Love-Related Emotion Intensity Scale; MBI = Maslach Burnout Inventory; EE = Emotional Exhaustion; DEP = Depersonalization; PA = Personal Accomplishment; EPQ = Eysenck Personality Questionnaire; EXT = Extraversion; NEU = Neuroticism; PSY = Psychoticism; LIE = Lie Scale.

**Table 2:** Path model’s parameters (significant effects).

	b	SE	z-Value	*p*	95% C.I.		Std. All *	R^2^
					Lower	Upper		
LREIS								0.058
EXT	0.515	0.117	4.389	<0.001	0.285	0.746	0.218	
LES								0.063
EXT	0.773	0.166	4.670	<0.001	0.449	1.098	0.231	
SCBCS								0.118
EXT	0.279	0.057	4.891	<0.001	0.167	0.391	0.235	
NEU	0.131	0.054	2.408	0.016	0.024	0.238	0.123	
LIE	0.268	0.070	3.840	<0.001	0.131	0.405	0.190	
Gender	−1.796	0.610	−2.943	0.003	−2.992	−0.600	−0.139	
EE								0.319
NEU	1.292	0.116	11.147	<0.001	1.065	1.519	0.504	
Age	0.141	0.050	2.826	0.005	0.043	0.238	0.123	
Shift Type	3.339	0.999	3.343	0.001	1.381	5.297	0.140	
DEP								0.257
LREIS	0.064	0.031	2.021	0.043	0.002	0.125	0.100	
SCBCS	−0.165	0.061	−2.683	0.007	−0.285	−0.044	−0.130	
NEU	0.358	0.064	5.605	<0.001	0.233	0.483	0.265	
PSY	0.729	0.140	5.215	<0.001	0.455	1.003	0.239	
Shift Type	1.623	0.550	2.949	0.003	0.544	2.702	0.129	
PA								0.278
LREIS	0.107	0.033	3.275	0.001	0.043	0.170	0.160	
SCBCS	0.287	0.064	4.524	<0.001	0.163	0.412	0.216	
NEU	−0.318	0.066	−4.803	<0.001	−0.447	−0.188	−0.224	
Gender	−1.607	0.758	−2.120	0.034	−3.092	−0.122	−0.093	
Age	0.125	0.028	4.400	<0.001	0.069	0.181	0.198	

Note. * Total Standardized Solution; LES = Love Experience Scale; LREIS = Love-Related Emotion Intensity Scale; SCBCS = Santa Clara Brief Compassion Scale; EE = Emotional Exhaustion; DEP = Depersonalization; PA = Personal Accomplishment; EXT = Extraversion; NEU = Neuroticism; PSY = Psychoticism; LIE = Lie Scale; b = unstandardized path coefficient; SE = standard error; Std. All = standardized path coefficient; R^2^ = proportion of variance explained for each endogenous variable.

**Table 3:** Significant indirect and/or total effects through compassion and love scales.

		b	SE	z-Value	*p*	95% C.I.		Std. All *
						Lower	Upper	
EE through SCBCS								
NEU	Indir.	−0.005	0.015	−0.333	0.739	−0.034	0.024	−0.002
	**Total**	**1.287**	**0.115**	**11.197**	**<0.001**	**1.062**	**1.513**	**0.502**
DEP through SCBCS								
EXT	**Indir.**	**−0.046**	**0.02**	**−2.352**	**0.019**	**−0.084**	**−0.008**	**−0.03**
	Total	−0.112	0.07	−1.607	0.108	−0.249	0.025	−0.075
NEU	Indir.	−0.022	0.012	−1.792	0.073	−0.045	0.002	−0.016
	**Total**	**0.383**	**0.069**	**5.584**	**<0.001**	**0.249**	**0.518**	**0.273**
PSY	Indir.	0.025	0.022	1.156	0.248	−0.017	0.068	0.008
	**Total**	**0.754**	**0.141**	**5.348**	**<0.001**	**0.478**	**1.031**	**0.248**
LIE	**Indir.**	**−0.044**	**0.02**	**−2.199**	**0.028**	**−0.083**	**−0.005**	**−0.025**
	Total	−0.162	0.084	−1.928	0.054	−0.327	0.003	−0.091
Gender	**Indir.**	**0.296**	**0.149**	**1.982**	**0.047**	**0.003**	**0.588**	**0.018**
	**Total**	**1.707**	**0.727**	**2.347**	**0.019**	**0.282**	**3.132**	**0.104**
PA through SCBCS								
EXT	**Indir.**	**0.08**	**0.024**	**3.321**	**0.001**	**0.033**	**0.127**	**0.051**
	**Total**	**0.169**	**0.074**	**2.298**	**0.022**	**0.025**	**0.313**	**0.107**
NEU	**Indir.**	**0.038**	**0.018**	**2.126**	**0.034**	**0.003**	**0.072**	**0.027**
	**Total**	**−0.28**	**0.067**	**−4.153**	**<0.001**	**−0.412**	**−0.148**	**−0.197**
LIE	**Indir.**	**0.077**	**0.026**	**2.927**	**0.003**	**0.025**	**0.129**	**0.041**
	Total	0.134	0.089	1.509	0.131	−0.04	0.307	0.071
Gender	**Indir.**	**−0.516**	**0.209**	**−2.467**	**0.014**	**−0.926**	**−0.106**	**−0.03**
	**Total**	**−2.123**	**0.766**	**−2.772**	**0.006**	**−3.624**	**−0.622**	**−0.123**
DEP through LREIS								
EXT	Indir.	0.033	0.018	1.836	0.066	−0.002	0.068	0.022
	**Total**	**0.096**	**0.048**	**1.997**	**0.046**	**0.002**	**0.191**	**0.122**
PA through LREIS								
EXT	**Indir.**	**0.055**	**0.021**	**2.625**	**0.009**	**0.014**	**0.096**	**0.035**
	**Total**	**0.162**	**0.051**	**3.174**	**0.002**	**0.062**	**0.261**	**0.194**
NEU	Indir.	−0.004	0.012	−0.35	0.726	−0.028	0.019	−0.003
	**Total**	**0.102**	**0.033**	**3.059**	**0.002**	**0.037**	**0.168**	**0.157**
PSY	Indir.	−0.02	0.027	−0.736	0.461	−0.073	0.033	−0.006
	**Total**	**0.087**	**0.037**	**2.329**	**0.02**	**0.014**	**0.16**	**0.153**
LIE	Indir.	0.006	0.015	0.366	0.714	−0.025	0.036	0.003
	**Total**	**0.112**	**0.038**	**2.989**	**0.003**	**0.039**	**0.186**	**0.163**

Note. * Total Standardized Solution SCBCS = Santa Clara Brief Compassion Scale; LREIS = Love-Related Emotion Intensity Scale; EE = Emotional Exhaustion; DEP = Depersonalization; PA = Personal Accomplishment; EXT = Extraversion; NEU = Neuroticism; PSY = Psychoticism; LIE = Lie Scale; b = unstandardized path coefficient; SE = standard error; C.I. = confidence interval; Std. All = standardized path coefficient. Indirect effects represent mediation through SCBCS, LES, or LREIS; total effects include both direct and indirect influences. The bold values indicate statistically significant results.

## Data Availability

The data presented in this study are available on request from the corresponding author due to privacy and confidentiality restrictions.

## Supplementary Materials

The following supporting information can be downloaded at: https://www.mdpi.com/article/10.3390/healthcare14030404/s1, Table S1: Nested path model comparisons (chi-square difference tests); Table S2: Full path model parameter estimates; Table S3: Full indirect and total effects through compassion and love-related emotion intensity; Figure S1: Partial correlations between personality traits and burnout dimensions controlling for compassion and love-related variables.

## Abbreviations

The following abbreviations are used in this manuscript:

EE	Emotional Exhaustion
DEP	Depersonalization
PA	Personal Accomplishment
MBI	Maslach Burnout Inventory
EPQ	Eysenck Personality Questionnaire
SCBCS	Santa Clara Brief Compassion Scale
LES	Love Experience Scale
LREIS	Love-Related Emotion Intensity Scale
RMSEA	Root Mean Square Error of Approximation
TLI	Tucker–Lewis Index
CFI	Comparative Fit Index
SRMR	Standardized Root Mean Square Residual
SD	Standard Deviation
χ^2^	Chi-square
Δχ^2^	Chi-square difference
b	unstandardized regression coefficient
*p*	*p*-value
EXT	Extraversion
NEU	Neuroticism
PSY	Psychoticism
LIE	Lie scale
R	Statistical computing language
df	Degrees of freedom
