# Supplementary material for: Love, Compassion, and Personality as Predictors of Burnout in Nurses: A Path Analysis Study
Source: Healthcare (Basel). 2026 Feb 5;14(3):404. doi: 10.3390/healthcare14030404 (PMC12898037; doi:10.3390/healthcare14030404)
Supplement: Supplementary file 1 [file healthcare-14-00404-s001.zip › healthcare-4076315-supplementary.pdf]

Supplementary Materials

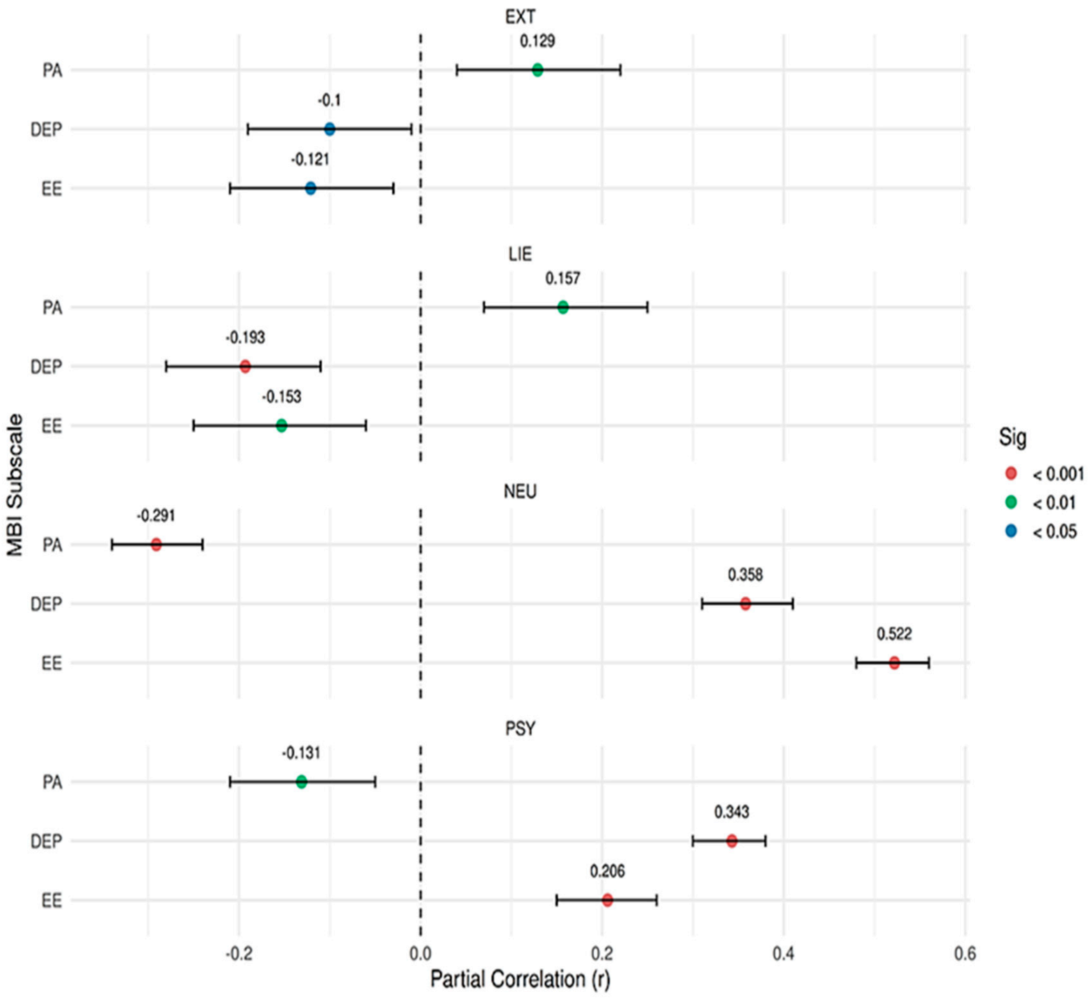

Figure S1. Partial Correlations between Personality Traits and Burnout Dimensions Controlling for Compassion and Love-Related Variables

Table S1. Nested Path Model Comparisons (Chi-Square Difference Tests)

	df	AIC	BIC	c^2	Δc^2	RMSEA	Δdf	p
Full Model	6	16485	16713	5.843				
Only LES or LREIS as mediator. Comparison with full model								
L1: LES as mediator	14	16514	16710	50.218	44.375	0.106	8	< 0.001
L2: LREIS as mediator	14	16501	16697	37.469	31.626	0.086	8	< 0.001
No love variable as mediator. Comparison with L1 and L2								
Comparison with L1	22	16519	16683	71.793	21.575	0.065	8	0.006
Comparison with L2					34.324	0.090	8	< 0.001

Note: LES = Love Experience Scale; LREIS = Love-Related Emotion Intensity Scale

Table S2. Path model's parameters

	b	SE	z-value	p	95% C.I.		Std. all*	R^2
					Lower	Upper		
LREIS								0.058
**EXT**	**0.515**	**0.117**	**4.389**	**< 0.001**	**0.285**	**0.746**	**0.218**	
NEU	-0.039	0.112	-0.352	0.725	-0.259	0.180	-0.019	
PSY	-0.186	0.246	-0.756	0.450	-0.668	0.296	-0.039	
LIE	0.053	0.144	0.368	0.713	-0.229	0.335	0.019	
Gender	1.963	1.257	1.561	0.118	-0.501	4.427	0.076	
LES								0.063
**EXT**	**0.773**	**0.166**	**4.670**	**< 0.001**	**0.449**	**1.098**	**0.231**	
NEU	0.018	0.158	0.113	0.910	-0.292	0.328	0.006	
PSY	-0.462	0.347	-1.332	0.183	-1.141	0.218	-0.068	
LIE	-0.066	0.203	-0.326	0.744	-0.464	0.331	-0.017	
Gender	3.003	1.772	1.694	0.090	-0.471	6.477	0.083	
SCBCS								0.118
**EXT**	**0.279**	**0.057**	**4.891**	**< 0.001**	**0.167**	**0.391**	**0.235**	
**NEU**	**0.131**	**0.054**	**2.408**	**0.016**	**0.024**	**0.238**	**0.123**	
PSY	-0.153	0.119	-1.281	0.200	-0.387	0.081	-0.064	
**LIE**	**0.268**	**0.070**	**3.840**	**< 0.001**	**0.131**	**0.405**	**0.190**	

**Gender**	**-1.796**	**0.610**	**-2.943**	**0.003**	**-2.992**	**-0.600**	**-0.139**	
EE								0.319
LREIS	-0.044	0.057	-0.768	0.443	-0.156	0.068	-0.036	
LES	-0.036	0.039	-0.916	0.360	-0.114	0.041	-0.042	
SCBCS	-0.037	0.111	-0.337	0.736	-0.256	0.181	-0.016	
EXT	-0.078	0.127	-0.614	0.539	-0.327	0.171	-0.027	
**NEU**	**1.292**	**0.116**	**11.147**	**< 0.001**	**1.065**	**1.519**	**0.504**	
PSY	0.414	0.254	1.630	0.103	-0.084	0.911	0.072	
LIE	-0.081	0.154	-0.526	0.599	-0.383	0.221	-0.024	
Gender	1.236	1.328	0.930	0.352	-1.368	3.839	0.040	
**Age**	**0.141**	**0.050**	**2.826**	**0.005**	**0.043**	**0.238**	**0.123**	
**Shift Type**	**3.339**	**0.999**	**3.343**	**0.001**	**1.381**	**5.297**	**0.140**	
DEP								0.257
**LREIS**	**0.064**	**0.031**	**2.021**	**0.043**	**0.002**	**0.125**	**0.100**	
LES	-0.041	0.022	-1.891	0.059	-0.084	0.002	-0.092	
**SCBCS**	**-0.165**	**0.061**	**-2.683**	**0.007**	**-0.285**	**-0.044**	**-0.130**	
EXT	-0.066	0.070	-0.950	0.342	-0.203	0.071	-0.044	
**NEU**	**0.358**	**0.064**	**5.605**	**< 0.001**	**0.233**	**0.483**	**0.265**	
**PSY**	**0.729**	**0.140**	**5.215**	**< 0.001**	**0.455**	**1.003**	**0.239**	
LIE	-0.118	0.085	-1.391	0.164	-0.285	0.048	-0.066	

Gender	1.411	0.732	1.928	0.054	-0.024	2.846	0.086	
Age	0.001	0.027	0.039	0.969	-0.053	0.055	0.002	
**Shift Type**	**1.623**	**0.550**	**2.949**	**0.003**	**0.544**	**2.702**	**0.129**	
PA								0.278
**LREIS**	**0.107**	**0.033**	**3.275**	**0.001**	**0.043**	**0.170**	**0.160**	
LES	0.035	0.023	1.573	0.116	-0.009	0.080	0.075	
**SCBCS**	**0.287**	**0.064**	**4.524**	**< 0.001**	**0.163**	**0.412**	**0.216**	
EXT	0.089	0.072	1.229	0.219	-0.053	0.231	0.056	
**NEU**	**-0.318**	**0.066**	**-4.803**	**< 0.001**	**-0.447**	**-0.188**	**-0.224**	
PSY	-0.085	0.145	-0.589	0.556	-0.369	0.199	-0.027	
LIE	0.057	0.088	0.645	0.519	-0.116	0.229	0.030	
**Gender**	**-1.607**	**0.758**	**-2.120**	**0.034**	**-3.092**	**-0.122**	**-0.093**	
**Age**	**0.125**	**0.028**	**4.400**	**< 0.001**	**0.069**	**0.181**	**0.198**	
Shift Type	0.073	0.570	0.127	0.899	-1.044	1.189	0.005	

* Total Standardized Solution SCBCS = Santa Clara Brief Compassion Scale; LES = Love Experience Scale; LREIS = Love-Related Emotion Intensity Scale; EE = Emotional Exhaustion; DEP = Depersonalization; PA = Personal Accomplishment; EXT = Extraversion; NEU = Neuroticism; PSY = Psychoticism; LIE = Lie Scale; b = unstandardized path coefficient; SE = standard error; CI = confidence interval; Std. all = standardized path coefficient

Table S3. Path model's parameters Indirect and total effects through compassion and love

		b	SE	z-value	p	95% C.I. Lower	95% C.I. Upper	Std. all
EE through SCBCS								
EXT	Indir.	-0.01	0.031	-0.336	0.737	-0.071	0.051	-0.004
	Total	-0.088	0.126	-0.703	0.482	-0.335	0.158	-0.031
NEU	Indir.	-0.005	0.015	-0.333	0.739	-0.034	0.024	-0.002
	**Total**	**1.287**	**0.115**	**11.197**	**< 0.001**	**1.062**	**1.513**	**0.502**
PSY	Indir.	0.006	0.018	0.326	0.745	-0.029	0.04	0.001
	Total	0.419	0.254	1.654	0.098	-0.078	0.916	0.073
LIE	Indir.	-0.01	0.03	-0.335	0.737	-0.069	0.049	-0.003
	Total	-0.091	0.151	-0.602	0.547	-0.388	0.206	-0.027
Gender	Indir.	0.067	0.201	0.334	0.738	-0.327	0.462	0.002
	Total	1.303	1.307	0.997	0.319	-1.259	3.864	0.042
DEP through SCBCS								
EXT	**Indir.**	**-0.046**	**0.02**	**-2.352**	**0.019**	**-0.084**	**-0.008**	**-0.03**
	Total	-0.112	0.07	-1.607	0.108	-0.249	0.025	-0.075
NEU	Indir.	-0.022	0.012	-1.792	0.073	-0.045	0.002	-0.016

	**Total**	**0.383**	**0.069**	**5.584**	**< 0.001**	**0.249**	**0.518**	**0.273**
PSY	Indir.	0.025	0.022	1.156	0.248	-0.017	0.068	0.008
	**Total**	**0.754**	**0.141**	**5.348**	**< 0.001**	**0.478**	**1.031**	**0.248**
LIE	**Indir.**	**-0.044**	**0.02**	**-2.199**	**0.028**	**-0.083**	**-0.005**	**-0.025**
	Total	-0.162	0.084	-1.928	0.054	-0.327	0.003	-0.091
Gender	**Indir.**	**0.296**	**0.149**	**1.982**	**0.047**	**0.003**	**0.588**	**0.018**
	**Total**	**1.707**	**0.727**	**2.347**	**0.019**	**0.282**	**3.132**	**0.104**
PA through SCBCS								
EXT	**Indir.**	**0.08**	**0.024**	**3.321**	**0.001**	**0.033**	**0.127**	**0.051**
	**Total**	**0.169**	**0.074**	**2.298**	**0.022**	**0.025**	**0.313**	**0.107**
NEU	**Indir.**	**0.038**	**0.018**	**2.126**	**0.034**	**0.003**	**0.072**	**0.027**
	**Total**	**-0.28**	**0.067**	**-4.153**	**< 0.001**	**-0.412**	**-0.148**	**-0.197**
PSY	Indir.	-0.044	0.036	-1.233	0.218	-0.114	0.026	-0.014
	Total	-0.129	0.149	-0.869	0.385	-0.42	0.162	-0.04
LIE	**Indir.**	**0.077**	**0.026**	**2.927**	**0.003**	**0.025**	**0.129**	**0.041**
	Total	0.134	0.089	1.509	0.131	-0.04	0.307	0.071
Gender	**Indir.**	**-0.516**	**0.209**	**-2.467**	**0.014**	**-0.926**	**-0.106**	**-0.03**
	**Total**	**-2.123**	**0.766**	**-2.772**	**0.006**	**-3.624**	**-0.622**	**-0.123**
EE through LES								

EXT	Indir.	-0.028	0.031	-0.899	0.369	-0.089	0.033	-0.01
	Total	-0.064	0.07	-0.913	0.361	-0.202	0.074	-0.052
NEU	Indir.	-0.001	0.006	-0.112	0.911	-0.012	0.011	0
	Total	-0.037	0.041	-0.907	0.365	-0.116	0.043	-0.043
PSY	Indir.	0.017	0.022	0.755	0.45	-0.027	0.06	0.003
	Total	-0.019	0.025	-0.789	0.43	-0.068	0.029	-0.04
LIE	Indir.	0.002	0.008	0.307	0.759	-0.013	0.018	0.001
	Total	-0.034	0.038	-0.898	0.369	-0.107	0.04	-0.042
Gender	Indir.	-0.109	0.135	-0.806	0.42	-0.373	0.156	-0.004
	Total	-0.145	0.171	-0.849	0.396	-0.479	0.19	-0.046
DEP through LES								
EXT	Indir.	-0.032	0.018	-1.753	0.08	-0.067	0.004	-0.021
	Total	-0.073	0.039	-1.862	0.063	-0.15	0.004	-0.113
NEU	Indir.	-0.001	0.007	-0.113	0.91	-0.014	0.012	-0.001
	Total	-0.022	0.018	-1.2	0.23	-0.058	0.014	-0.085
PSY	Indir.	0.019	0.017	1.089	0.276	-0.015	0.053	0.006
	Total	-0.022	0.018	-1.2	0.23	-0.058	0.014	-0.085
LIE	Indir.	0.003	0.008	0.321	0.748	-0.014	0.019	0.002
	Total	-0.038	0.022	-1.749	0.08	-0.082	0.005	-0.09

Gender	Indir.	-0.124	0.098	-1.262	0.207	-0.316	0.068	-0.008
	Total	-0.165	0.114	-1.45	0.147	-0.388	0.058	-0.099
PA through LES								
EXT	Indir.	0.027	0.018	1.491	0.136	-0.009	0.063	0.017
	Total	0.063	0.04	1.556	0.12	-0.016	0.142	0.092
NEU	Indir.	0.001	0.006	0.113	0.91	-0.01	0.012	0
	Total	0.036	0.024	1.528	0.126	-0.01	0.082	0.075
PSY	Indir.	-0.016	0.016	-1.016	0.309	-0.048	0.015	-0.005
	Total	0.019	0.017	1.105	0.269	-0.015	0.053	0.07
LIE	Indir.	-0.002	0.007	-0.319	0.749	-0.017	0.012	-0.001
	Total	0.033	0.022	1.489	0.137	-0.01	0.077	0.074
Gender	Indir.	0.106	0.092	1.153	0.249	-0.075	0.287	0.006
	Total	0.142	0.11	1.291	0.197	-0.074	0.357	0.081
EE through LREIS								
EXT	Indir.	-0.023	0.03	-0.756	0.449	-0.081	0.036	-0.008
	Total	-0.066	0.087	-0.767	0.443	-0.236	0.103	-0.044
NEU	Indir.	0.002	0.005	0.32	0.749	-0.009	0.012	0.001
	Total	-0.042	0.055	-0.765	0.444	-0.15	0.066	-0.036
PSY	Indir.	0.008	0.015	0.539	0.59	-0.021	0.038	0.001

	Total	-0.036	0.048	-0.748	0.454	-0.129	0.058	-0.035
LIE	Indir.	-0.002	0.007	-0.332	0.74	-0.016	0.011	-0.001
	Total	-0.046	0.06	-0.764	0.445	-0.165	0.072	-0.037
Gender	Indir.	-0.086	0.125	-0.689	0.491	-0.331	0.159	-0.003
	Total	-0.13	0.178	-0.73	0.465	-0.478	0.219	-0.039
DEP through LREIS								
EXT	Indir.	0.033	0.018	1.836	0.066	-0.002	0.068	0.022
	**Total**	**0.096**	**0.048**	**1.997**	**0.046**	**0.002**	**0.191**	**0.122**
NEU	Indir.	-0.003	0.007	-0.347	0.729	-0.017	0.012	-0.002
	Total	0.052	0.03	1.725	0.084	-0.007	0.111	0.096
PSY	Indir.	-0.012	0.017	-0.708	0.479	-0.044	0.021	-0.004
	Total	0.052	0.03	1.725	0.084	-0.007	0.111	0.096
LIE	Indir.	0.003	0.009	0.362	0.717	-0.015	0.022	0.002
	Total	0.067	0.034	1.948	0.051	0	0.134	0.102
Gender	Indir.	0.125	0.101	1.236	0.217	-0.073	0.323	0.008
	Total	0.188	0.123	1.534	0.125	-0.052	0.429	0.108
PA through LREIS								
EXT	**Indir.**	**0.055**	**0.021**	**2.625**	**0.009**	**0.014**	**0.096**	**0.035**
	**Total**	**0.162**	**0.051**	**3.174**	**0.002**	**0.062**	**0.261**	**0.194**

NEU	Indir.	-0.004	0.012	-0.35	0.726	-0.028	0.019	-0.003
	**Total**	**0.102**	**0.033**	**3.059**	**0.002**	**0.037**	**0.168**	**0.157**
PSY	Indir.	-0.02	0.027	-0.736	0.461	-0.073	0.033	-0.006
	**Total**	**0.087**	**0.037**	**2.329**	**0.02**	**0.014**	**0.16**	**0.153**
LIE	Indir.	0.006	0.015	0.366	0.714	-0.025	0.036	0.003
	**Total**	**0.112**	**0.038**	**2.989**	**0.003**	**0.039**	**0.186**	**0.163**
Gender	Indir.	0.209	0.148	1.409	0.159	-0.082	0.5	0.012
	Total	0.316	0.165	1.913	0.056	-0.008	0.639	0.172

*Note: SCBCS = Santa Clara Brief Compassion Scale; EE = Emotional Exhaustion; DEP = Depersonalization; PA = Personal Accomplishment; EXT = Extraversion; NEU = Neuroticism; PSY = Psychoticism; LIE = Lie Scale; b = unstandardized path coefficient; SE = standard error; CI = confidence interval; Std. all = standardized path coefficient
